# A288 PHYSICIAN PERSPECTIVES OF MALNUTRITION CARE IN NOVA SCOTIA: A MORE-2-EAT (M2E) NOVA SCOTIA PHASE 2 QUALITATIVE SUBSTUDY

**DOI:** 10.1093/jcag/gwac036.288

**Published:** 2023-03-07

**Authors:** D Veldhuijzen van Zanten, L Gramlich, L Cahill

**Affiliations:** 1 University of Alberta; 2 Unviersity of Alberta, Edmonton; 3 Dalhousie University, Halifax, Canada

## Abstract

**Background:**

Malnutrition is a clinical entity that is often underdiagnosed due to a lack of standard nutrition status screening during patient encounters, a lack of malnutrition education throughout medical training, inadequate resources to provide proper nutritional care, and differing perceptions regarding the importance of this condition. Amongst patients whose malnutrition is left untreated, approximately two thirds of them will experience further decline in their nutritional status. It is therefore important that the nutritional status of each patient be evaluated with every healthcare encounter where malnutrition may be relevant.

**Purpose:**

The purpose of this study is to assess generalist and specialist physician perspectives on patient malnutrition care at various Nova Scotia healthcare sites. The aim is to better understand physician viewpoints and attitudes towards malnutrition and nutrition care, its role in their patient care, and what factors might facilitate or impede its effective use in clinical practice.

**Method:**

Using the Consolidated Framework of Implementation Research (CFIR) and Theoretical Domains Framework (TDF) approaches and ethics approval from the Nova Scotia Health Authority, individually recorded virtual interviews were conducted with physicians working in Nova Scotia. Standardized questions on malnutrition were asked to a target of 12-24 physicians Responses were transcribed and qualitatively analyzed using NVivo software by a professional data analyst.

**Result(s):**

To date, ten interviews have been completed. Preliminary qualitative analysis indicates that while all physicians agreed that malnutrition is an important aspect of a patient’s care, its consistent screening and ongoing management is variable. Key Theoretical Domains identified were Knowledge, Skills, Social/Professional Role and Identity, Beliefs about Capabilities, Memory Attention and Decisions Processes, and Social Influences. Overall, most physicians had a knowledge deficit related to nutrition care and malnutrition, which led to uncertainty on how to screen or manage it. Standardized malnutrition screening tools were infrequently used. This responsibility was often deferred to the dietitian, thereby minimizing the role that physicians play or believe they have in its management. A lack of time during clinical encounters was also a key contributing factor.

**Conclusion(s):**

Strategies to optimize physician involvement in and awareness of nutrition care include increased education to train physicians to recognize and manage malnutrition in patients. The use of health system resources such as standardized malnutrition screening tools when malnutrition may be an issue can help with earlier identification and subsequent management. In addition, a team-based approach consisting of healthcare professionals with knowledge of malnutrition that actively involves the physician may be the most appropriate way for patient malnutrition to be effectively managed and provide education to both the physician and the patient.

**Please acknowledge all funding agencies by checking the applicable boxes below:**

None

**Disclosure of Interest:**

D. Veldhuijzen van Zanten Shareholder of: N/A, Consultant of: N/A, Employee of: N/A, Paid Instructor of: N/A, Speakers bureau of: N/A, L. Gramlich Grant / Research support from: Baxter, research funds, principal investigator; Nestle, education funds, chair; and Fresenius Kabi, research funds, principal investigator., Consultant of: Abbott, honorarium for consultant/advisory board, L. Cahill Grant / Research support from: Canadian Institutes of Health Research (CIHR), Research Nova Scotia, Nova Scotia Health Research Fund, Dalhousie University’s Internal Medicine Research Fund (UIMRF), Nova Scotia Health Research Foundation, Queen Elizabeth II Foundation.

